# Determining the Actual Prevalence of Hepatitis B in Khyber Pakhtunkhwa-Pakistan: A Meta-Analysis

**DOI:** 10.2174/1874357901812010033

**Published:** 2018-02-28

**Authors:** Najeeb Ullah Khan, Ali Zalan, Arnolfo Petruzziello, Iftikhar ud din, Fazle Haq, Yousaf Hayat

**Affiliations:** 1 Institute of Biotechnology and Genetic Engineering (Health Division), The University of Agriculture Peshawar, Peshawar, Pakistan; 2 Department of Mathematics, Stats & Computer Science, The University of Agriculture Peshawar, Peshawar, Pakistan; 3 Virology and Molecular Biology Unit “V. Tridente”, Istituto Nazionale Tumori - Fondazione “G. Pascale”, IRCCS Italia, Naples, Italy

**Keywords:** Hepatitis B, Liver, Prevalence, Meta analyze, Infection, Healthcare

## Abstract

**Background:**

Hepatitis B is considered the most dangerous among the five types of Hepatitis, as it is clinically asymptomatic. It can silently damage the liver over many years without being diagnosed. Hepatitis B is one of the top risks of liver complications in Khyber Pakhtunkhwa (KP), a province of Pakistan, with an average prevalence rate of 2.70%.

**Aims:**

We aimed to carefully review the previously published data on prevalence of Hepatitis B Virus (HBV) in KP-Pakistan and use the statistical approach to obtain more precise estimate of the prevalence of HBV in KP-Pakistan. This study on one hand will provide a more reliable and consolidated estimate (pooled estimate) of HBV in the stated region, on the other hand, it enabled us to judge the heterogeneity among the estimates found from these studies. The study is intended to provide more authentic prevalence record and help government/ non-government organizations and health professionals, which plan to initiate HBV prevention programs in KP-Pakistan.

**Methods:**

A meta-analysis was performed based on studies found in literature search from electronic databases and bibliography on the prevalence of HBV in KP-Pakistan from 2007 to 2017. Abstracts and results of twenty papers were thoroughly studied and the data were extracted. The findings from these studies were distributed in two groups (general and population at high risk) constituting 15 and 5 studies respectively.

**Results:**

The combined prevalence by considering random model for the general population of KP-Pakistan was observed to be 2.71%, while population at high risk was reasonably high *i.e*. 5.64%. By comparing this prevalence rate to the highest global prevalence of HBV in the adult population of Western Pacific Region (6.2%), significant (*p*-value= 0.000) heterogeneity was observed among the estimates in each group. However, the funnel plot provides a symmetric look, eliminating the effect of publication bias. We can say that HBV has an alarming prevalence rate in KP-Pakistan. However, HBV is thrice more prevalent in male population of KP-Pakistan than the female population.

**Conclusion:**

The above results lead that HBV infection has reached an alarming state in KP-Pakistan, though projects like Prime Minister’s Program for Prevention & Control of Hepatitis which are contributing in improving the health of the people of KP by trying to prevent and control the incidence of HBV. More massive vaccination and awareness programs should be initiated to prevent the spread of HBV on urgent basis. Provision of diagnostics and treatment facilities against HBV in healthcare units of KP-Pakistan should be assured.

## 1 INTRODUCTION

Hepatitis B is a deadly liver infection, which is caused by the Hepatitis B Virus (HBV). Approximately, 257 million people are living with HBV infection that resulted in 887,000 deaths in 2015 [1]. The highest prevalence of HBV was reported in the adult population of Western Pacific Region (6.2%) and African Region (6.1%) [2, 3]. HBV infection can result in many liver complications including chronic hepatitis, cirrhosis, and hepatocellular carcinoma [4]. The major risk factors for HBV are the unhygienic and substandard methods of surgeries, use of unsterilized instruments, tattooing, piercing, blood transfusion and the drugs abuse. The risk factors for developing HBV infection also include working in a healthcare setting, dialysis, acupuncture, tattooing, sharing razors or toothbrushes with an infected person, travelling in countries where HBV is endemic, and residence in an institution [5].

HBV is endemic in Pakistan with prevalence rate as high as 3% [6]. High prevalence of HBV in Pakistan is observed in people who have a low economic status. It is vital to control HBV in Pakistan because 67.5% of the population belongs to rural areas of low economic status [7]. Effective treatments, mass immunization programs, and safe injection techniques are important for eliminating HBV infection. Effective vaccines against HBV are available since 1982 [8]. Adefovir is an effective prescription medicine used to treat infections with HBV [9]. Entecavir, sold under the brand name Baraclude, is an antiviral medication used in the treatment of HBV infection. It proves to be an emerging effective drug against the HBV [10].

The prevalence of HBV in Pakistan has been quoted about 3–5% in general population and about 10–20% in population at high risk [6]. Actual determination of HBV infection in Pakistan is difficult because of poor management and record keeping. With the aim to investigate the actual prevalence of HBV in KP-Pakistan, meta-analysis of published data from KP-Pakistan was performed. A total of twenty papers from 2007–2017 were selected dividing in two groups (general and population at high risk). The extracted data was statistically analyzed and compared with the world’s highest prevalence rate, indicating that the prevalence of HBV in KP-Pakistan is an alarming situation.

## 2 MATERIALS AND METHODS

### 2.1 Literature Searching Strategy

Studies of interest from 2007–2017 (up to 14^th^ July, 2017) were selected using search engine of the PubMed Database, Google scholar and the relevant search strategies (prevalence of HBV in KPK, prevalence of HBV in N.W.F.P, prevalence of HBV in Khyber Pakhtunkhwa). We have also searched the database of NCBI and Researchgate.net, by using the keywords [HBV in Khyber Pakhtunkhwa]. A total of 20 studies (abstracts and results) were thoroughly studied and selected for the analysis. The data (Table **1**) regarding, region, study population (general or high-risk), lab techniques used for markers detection, total sample size, percentages and numbers of HBV was identified and subjected for statistical analysis.

### 2.2 Data Synthesis and Meta-analysis

The advent of latest and sophisticated statistical tools enabled us to reanalyze the secondary type of data. Furthermore, different studies on the same topic may confusingly differ in their findings. One discipline, which considerably improves secondary analysis, is termed as meta-analysis. It is a known fact that the research activities are rapidly growing; there is an urgent need to integrate all the studies on the same topic. Meta-analysis refers to the statistical analysis of results obtained from individual studies, in order to come up with a combine and comprehensive result.

The collected studies were distributed into two groups, first group consist of 15 studies and were considered as representative of the general population, while the second group consists of 5 studies that represent the population at high risk. The meta-analysis tools of pooling the estimates, forest plot, heterogeneity testing and assessing publication bias were used. Further, 95% confidence intervals (CIs) and Z and *P* value of the respective event rates were obtained. The corresponding forest plot provided a better picture of event rates (prevalence when multiplied by 100) along with 95% CIs.

Next the combined estimate of these studies was found by considering both fixed and random effect model; in order to judge how much these studies are in accordance with each other, we used the heterogeneity test (I-square). To assess the biasness, symmetry and divergence of the results from respective studies, a funnel plot was used.

## 3 RESULTS

### 3.1 Statistical Analysis

A total of 20 articles were identified through searching records and included in the study after screening the titles and abstracts. The characteristics of the studies are summarized (Table **1**). The HBV prevalence rate in the studies, which represents the general population varies from 1.10% of blood donors to 21.1% of the internally displaced population (IDPs). The combined estimate of the general population based on 15 studies was 2.8% (CI 2.7% to 2.9%), which dropped to 2.3% when a study (IDPs) was excluded and consequently the prevalence in the risk group (based on 6 studies) rose to 6.1%. All the assessments included in the study showed significance prevelance rate of HBV in the respective population. The forest plot (Table **2**) gives a better view of the estimates along with their CIs. It is evident that apart from two studies i.e. one of IDPs and the drug addicts, where the prevalence was unusually high (21.1% and 22.4% respectively), the corresponding CIs was large too, the rest of the studies showed more conformity and precision of estimates.

In the computation of general population estimate, the study of blood donor C constituted 32042 individuals and consequently attained maximum weight (34.11%). It was followed by the study based on HBV in students and employees, which obtained 12.98% weightage. In the high risk category, the prevalence of the hospital patients reported extra ordinary high weightage of 88.84% while the remaining 11.16% was distributed among the remaining 4 studies.

### 3.2 Pooled Estimates for Fixed and Random Model

The combined prevalence of the fixed effect for the general population of KP was 2.80%. This estimate is highly significant (*p*-value= 0.00) and carries huge precision as indicated by the width of 95% CIs (2.68 to 2.93). Further, the result of the Q test indicates that the population of 15 studies was shown to possess highly significant heterogeneity (*p*-value= 0.00). The high *I*^2^ values (exceeding 75%) showed that most of the variability across studies is due to heterogeneity rather than chance, reflecting the inconsistency between the studies. Hence it will be appropriate to consider mixed or random effect model instead of fixed effect model.

The combined fixed effect estimate of group 2 populations was reasonably high as 5.25%, having greater width of 95% CIs (4.99 to 5.55%). The heterogeneity among 5 studies was observed to be highly significant as reflected by the corresponding score of Q-test and the relevant *p*-value (0.00). The estimated prevalence rate of overall fixed effect of the two groups was 3.71% with reasonable precision of 95% CIs (3.59 to 3.83%). The corresponding results from Z and Q-test were highly significant.

When considering mixed (random) effect model, the combined estimates of the prevalence rate were observed to be bit lower than the fixed effect (2.71%) with 95% CIs, ranging from 1.74 to 4.21. The prevalence rate was again significantly (*p*-value= 0.00) greater than the hypothesized value of zero. The population at higher risk now has a greater prevalence rate (5.64%) than before, with much wider CIs (2.99 to 10.35%) than the fixed effect estimate. The overall mixed effect estimate was calculated to be 3.44% but with least precision as the corresponding 95% CIs (2.39 to 4.91) was much wider than that found before. All these mixed effect estimates are statistically highly significant (Table **3**).

### 3.3 Assessment of Publication Bias

**Fig. (1):**
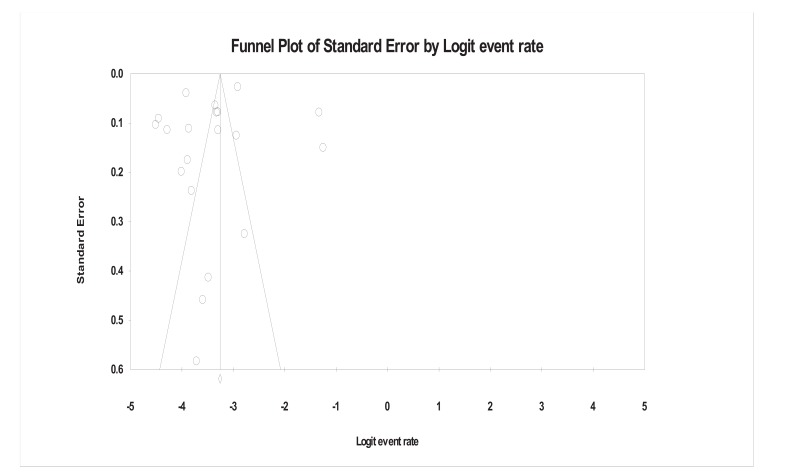
Funeral plot of overall 20 studies.

Although the event rates (prevalence) greatly vary, so does their standard error. Mostly (in 16 studies), the standard error is lower than 0.3. The funnel plot (Fig. **1**) of all the 20 studies shows that circles are scattered on both sides of the funnel but still, they were fairly symmetrical, a symptom that refers to no specification bias in the Meta-analysis we carried out. The line in the middle indicates the overall estimate obtained from the meta-analysis, which is the weighted mean of the studies included in the analysis.

### 3.4 Funnel Plot of the General and High-Risk Populations

**Fig. (2):**
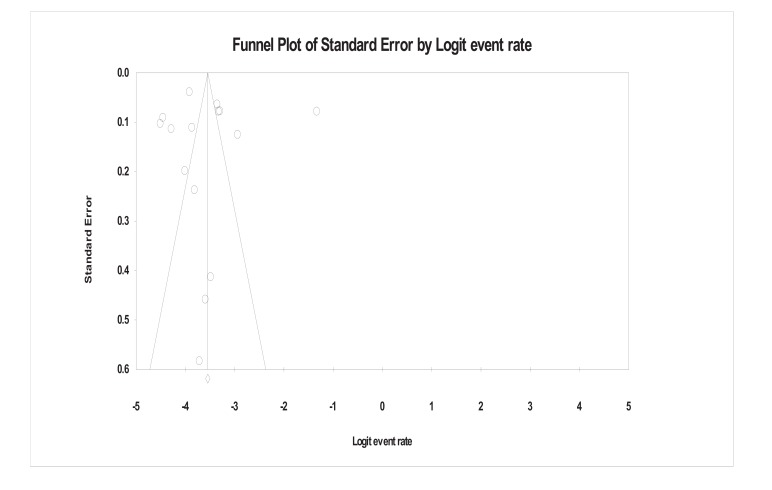
Funeral plot of group 1 studies.

**Fig. (3):**
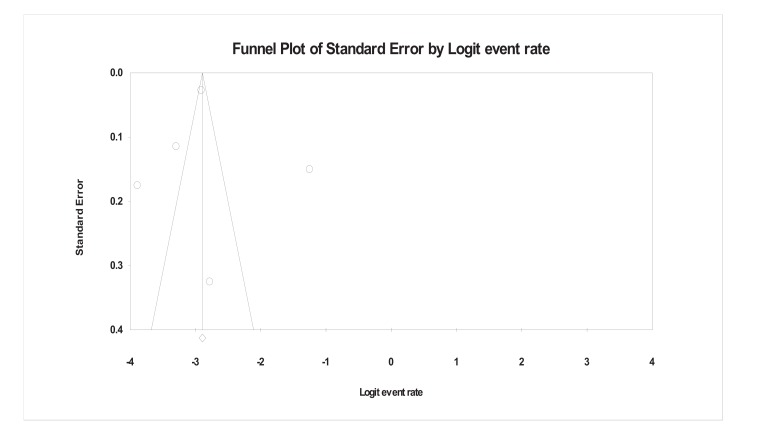
Funeral plot of group 2 studies.

Funnel plot of the general population (Fig. **2**) based on 15 studies depicts the same picture as stated above for all the 20 studies. Variations among the plotted dots were high but still the funnel depicted symmetrical shape referring to no publication bias. For the high-risk population (Fig. **3**), the doted points are widely scattered, reflecting the difference in standard error of these studies but still evenly distributed around the central line, giving symmetrical shape to the funnel plot thus no publication bias.

## 4 DISCUSSION

HBV is one of the major public health problems worldwide especially in developing countries including Pakistan [30]. HBV is clinically asymptomatic over many years after infection that can silently damage the liver, which leads to HCC [31]. Pakistan has a huge burden of HBV infection over many years with an average prevalence rate of 3-5% in general population and 10-20% in population with high risk [6]. Our objective was to document the actual prevalence of HBV in KP-Pakistan by reviewing the recent prevalence studies (2007 to 2017). The best way to analyze our data was to Meta analyze them so that we could come up with a more comprehensive result. The selected data were divided in to two groups; a general and a high-risk group. The combined prevalence of the fixed effect for the general population of KP was 2.80%, which doesn’t seem much high but the combined fixed effect estimate of the population at risk was reasonably high i.e. 5.25%. The high-risk group had drug abusers and other types of patients in it. The prevalence in drug abusers was found to be as high as 22.40% [27], which is quite alarming. Drug abuse places people at specific hazard for contracting viral hepatitis. Taking part in hazardous sexual conduct and drug abuse places, people get in danger of contracting HBV. They are at high hazard for contracting HBV from shared needles. On account of the addiction, drug abusers over and again take part in these dangerous practices, which can make them “super carriers” of the infection. In the group of general population, the IDPs had a prevalence rate as high as 21.05% [7]. Pakistan has been antagonistically influenced by the war on terror. It has caused calamity on an unfathomable scale. Pakistan, being the forefront state, has directed a number of operations against activists in the KP region. In the result of the military operation named Zarb-e-Azb, a great many individuals fled from their homes and incidentally relocated to safe territories to look for shelter [32]. The issues, which IDPs confront, are quick alleviation, protection, sustenance, medicine, clean drinking water, sanitation and so forth. These factors have led to the spread of lethal diseases like HBV. The government of KP must map IDPs in order to stop the spread of deadly diseases including HBV and the record proves that on their return, they would face and generate lots of health challenges.

In pregnant women, the percentage of prevalence (as recorded by two publications) was found to be 1.16% [12] and 1.37% [23]. The lowest prevalence 1.10% was found in the blood donors [11] but it will need further confirmation with huge numbers of subjects. KP has approximately high prevalence rate of 3.0% with a population of more than 30.52 million; KP has more than 10 million HBV carriers and contributes a large proportion to the worldwide pool of HBV carriers. It is vital to complete more examinations to better describe the disease transmission of HBV, for distinguishing high prevalence regions, and all the while to concentrate on enhancing general wellbeing measures to combat HBV and lessen its weight.

## CONCLUSION

Taking everything into account, there is a high weight of perpetual HBV virus among the residents of KP. HBV is a preventable disease and to be sure one that can be treated by immunization or vaccination. Tending to the difficulties of HBV in KP-Pakistan will require multi-level methodologies that incorporate populace wide instructive mediations and a fortifying of the Pakhtunkhwa’s wellbeing framework. There is pressing requirement for the legislature of KP and its universal accomplices to reorganize HBV control.

## Figures and Tables

**Table 1:** Characteristics summary of the included studies.

** Sample Size **	** Male **	** Female **	** HBV % **	** Male (+) **	** Female (+) **	** Region **	** Methods **	** HBV Marker **	**Reference**
8439	Irrespective of gender		1.10%			KPK	ELISA	HBsAg	[11]
10288		10288	1.16%		1194	Peshawar	ICT	HbsAg	[12]
2166	Irrespective of gender		3.60%			Peshawar	ELISA	HbsAg	[13]
180	144	36	2.70%	4	1	KPK	ICT/ ELISA	HbsAg	[14]
200	138	62	3.00%	4	2	Mardan	ICT	HbsAg	[15]
170	94	76	5.88%	7	3	Swat	ELISA	HbsAg	[16]
4639	3605	1034	3.59%	143	24	KPK	ICT/ELISA	HbsAg	[17]
824	493	331	2.18%	10	8	Peshawar	ICT	HbsAg	[18]
950	550	400	21.05%	157	43	Malakand Division	ELISA/ RT-PCR	HBsAg, Anti HBs, HBeAg, Anti HBe antibodies	[7]
3915	Irrespective of gender		2.07%			Peshawar	ICT	HBsAg	[19]
32,042	32042		1.97%	632		Peshawar	ELISA	HBsAg	[20]
7148	Irrespective of gender		3.41%			KPK & FATA	ICT/ELISA/RT-PCR	HBsAg	[21]
25944	13593	11991	5.20%	905	447	Bannu	ELISA	HBsAg	[22]
5607		5607	1.37%		77	Swat	ICT/ ELISA	HBsAg	[23]
1300	1214	86	5.07%	Not specified		Kurram Agency	ICT	HBsAg	[24]
4680	2870	1810	3.50%	100	64	Swat	ELISA	HBsAg	[25]
125	83	42	2.40%	2	1	Abbottabad	ELISA	HBsAg	[26]
252	252		22.40%	56		NWFP	ELISA	HBsAg	[27]
1418	872	544	1.80%	16	10	Abbottabad	ELISA	HBsAg	[28]
1630	1205	425	2.02%	28	5	Abbottabad	ELISA	HBsAg	[29]

**Table 2:** Overall statistics of the included studies.

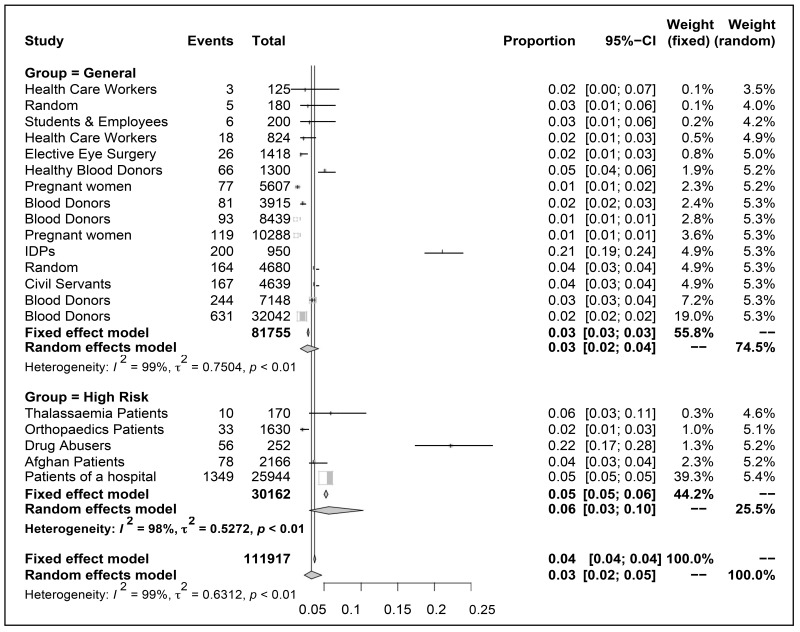

**Table 3:** Fixed effect analysis of overall, general and high risk groups.

Group		Effect Size and 95% CI			Test of Null (2-Tail)		Heterogeneity			
	No. of Studies	Point Estimate	Lower Limit	Upper Limit	Z-value	*P*-value	Q-value	df (Q)	*P*-value	I-Squared
**Fixed Effect Analysis**										
1	15	2.80	2.68	2.93	-151.01	0.00	1147.11	14	0.00	98.78
2	5	5.25	4.99	5.51	-109.79	0.00	162.83	4	0.00	97.54
Overall	20	3.71	3.59	3.83	-185.79	0.00	1650.65	19	0.00	98.85
**Mixed effects analysis of general, high-risk and overall populations**										
1	15	2.71	1.74	4.21	-15.48	0.00	–	–	–	–
2	5	5.64	2.99	10.35	-8.37	0.00	–	–	–	–
Overall	20	3.44	2.39	4.91	-17.50	0.00	–	–	–	–
